# A memory-theoretic account of citation propagation

**DOI:** 10.1098/rsos.231521

**Published:** 2024-05-29

**Authors:** Michael R. Dougherty, David A. Illingworth, Rosalind Nguyen

**Affiliations:** ^1^ Department of Psychology, University of Maryland, College Park, MD, USA; ^2^ Department of Psychology, California State University, Long Beach, CA, USA

**Keywords:** citation counts, memory, bibliometrics, research evaluation, information search

## Abstract

Despite the common assumption that citations are indicative of an article’s scientific merit, increasing evidence indicates that citation counts are largely driven by variables unrelated to quality. In this article, we treat people’s decisions of what to cite as an instance of memory retrieval and show that observed citation patterns are well accounted for by a model of memory. The proposed exposure model anticipates that small alterations in factors that affect people’s ability to retrieve to-be-cited articles from memory early in their life cycle are magnified over time and can lead to the emergence of highly cited papers. This effect occurs even when there is no variation in the starting point exposure probabilities (i.e. when assuming a level playing field where all articles are treated equally and of equal ‘quality’), and is exacerbated by natural variation in retrievability of articles due to encoding. We discuss the implications of the model within the context of research evaluation and hiring, tenure and promotion decisions.

## 1. Introduction

Recent research is concerned with how information propagates across information ecosystems. Of particular interest is the manner by which producers select information (from memory) to be shared and, thus, made available to the next receiver [1]. In this article, we develop a general model of information propagation and apply it to the domain of scholarly communication, in particular to modelling the distribution of citation patterns across 32 years. In what follows, we review background literature on citation behaviour implying that distributions of citation counts arise from cognitive processes that mimic an exposure-type mechanism, in which one decides which papers to cite in their own work based largely on context-dependent memory retrieval processes. We show that a simple model based on memory retrieval processes is sufficient for accounting for a number of findings in the bibliometric literature.

As a scientist, it is nice to know (or at least believe) that one’s work is being read and cited by others who find it useful. What makes an article highly cited, however, is of considerable debate. On one hand, citation counts are commonly treated as proxies for quality—the assumption being that a paper is more likely to be cited if it is of high quality. However intuitive it may seem to infer the reverse (i.e. that quality can be inferred from citation counts), the inference does not follow even though it seems reasonable to assert that quality might indeed drive some citations. The bibliometric literature indicates that there are many other factors unrelated to quality that also relate to citation counts. Posting an article to a preprint server [2], having a co-author whose name is well recognized [3], being part of a large academic social network [4], presenting at conferences [5] and the use of social media can all positively boost citation counts [6–9], while the inclusion of mathematical equations decreases citation rates [10]. There is even evidence that citations covary with length of titles and the use of colons and hyphens in titles [11,12]. Lurking underneath these factors is another curious finding: a relatively large proportion of citations are perfunctory—made for reasons that are not clearly necessary for the authors’ scientific arguments—while others reflect ‘negative’ endorsements [13,14]. Even more problematic is that authors cite articles without having read them or for the purpose of appeasing reviewers or editors [15].

The idea that citation counts are influenced by factors that are seemingly unrelated to quality is problematic for reasons related to how citation counts are used. It is no secret that counting the number of citations researchers garner figures prominently into people’s perceptions of scientific impact. These perceptions, founded on the belief that citations reflect quality, influence important decisions. The most obvious decisions are those related to hiring, tenure and promotion, wherein university promotion committees—from the department up to the president’s office—often want to see citation-based metrics. It is also likely that citation counts have other secondary effects, such as influencing speaker invitations, scientific awards, grant awards and even affecting university rankings. Such effects can, in principle, further impact citation behaviours by amplifying the exposure of those who receive invitations and awards and increasing the opportunity for their research to be viewed, while restricting opportunities for less well-cited authors. As we discuss later, these effects can have real-world practical implications that can adversely impact the career trajectories of some groups more than others.

The present work is broadly situated within both the bibliometric and cognitive literatures, but also directly relates to recent calls for reforming the research incentive system such as those proposed in the San Francisco Declaration on Research Assessment, the Coalition for Advancing Research Assessment, SCOPE, the Higher Education Leadership Initiative in Open Science and others [16,17], which currently relies heavily on metrics such as impact factors and citations. Several recent studies highlight disparities in citations across both racial and gender identities [18–20]. These studies illustrate the arbitrary nature of citation behaviours and point to one of the many problematic features of using citations in evaluative contexts—namely, that their use disadvantages women and underrepresented scholars.

Although it is tempting to maintain the belief that citation counts reflect some dimension of quality, the scientific literature examining this issue is equivocal. For instance, recent work has examined the relation between citation counts and objective measures of article quality such as the presence of inconsistencies in the reporting of statistics, the degree of evidential support provided by the data and replicability of scientific findings [21]. The revealed relations were weak and in some cases, citation counts were inversely related to quality. Other work has examined the relation between methodological soundness and citation counts and found no meaningful relation [22]. Similarly, analyses examining experts’ subjective ratings of quality often show weak or non-existent relations between rated quality and future citation counts [23–25]. In one recent analysis, for instance, researchers examined the relation between assessments of quality and citation counts in the field of chemistry [26]. In their study, the authors asked chemists to select three articles from an issue of *Journal of the American Chemical Society* that: (i) were the most significant, (ii) they believed to be the most highly cited, (iii) they would want to share with colleagues and (iv) they would want to share with the broader public. Correlations of actual citation count 13 years post-publication with judgments of significance ( r=0.19 ), which to share with others ( r=0.06 ) and which to share with the general public ( r=0.08 ) were all quite low.

Another often-referenced feature of high-quality research is 'novelty.’ Here too, when objective measures of novelty are defined, the relation with citation counts does not support their use as an index of quality. Novel research (that which makes unique connections across different literatures) accrues citations more slowly early in its life cycle than less novel work, meaning that work with greater novelty tends to be less well-cited early on post-publication [27]. Analysis of citation patterns 15 years post-publication indicates that citation counts for novel work have greater variability than less novel work: although citations to highly novel work is more likely to be in the top 10% 15 years post-publication, it is also more likely to be in the bottom 10% [27]. Finally, even one of the most thorough and extensive exercises in evaluating research quality, the Research Excellence Framework (REF) [28] failed to show consistent correlations between peer review and bibliometric indicators. Although the correlation between citation counts and REF quality scores was reasonably high in some disciplines, including psychology (e.g. Spearman’s rho =0.41 ), all reviewers had access to journal names and some review panels (e.g. psychology) had access to citation counts. It is reasonable to assume that reviewers are knowledgeable enough to know the impact factors of many of the journals. Indeed, the average correlation between citation counts and REF quality scores was substantially higher for panels with access to citation data (rho =0.50 ) compared with panels without (rho =0.17 , Bayes factor =85313 ).

On the flip side, analyses of duplicate publication of identical papers (i.e. papers published twice in different journals) reveal that citation counts for articles that are objectively equivalent in quality (because they are identical) do vary, primarily as a function of the journal impact factor [29]. Altogether, citation counts do not meaningfully vary as a function of objective indices of quality or subjective judgments of quality but do vary systematically within articles that are identical in quality. Coupled with the factors reviewed above that predict citation counts, these findings suggest that the link between quality and citation counts is tenuous at best [30].

Setting aside the factors reviewed above that affect citation counts, might there be another compelling yet parsimonious explanation for why some articles are cited a lot and others are not? In this article, we address this question by treating citation distributions as memory-theoretic phenomena that manifest as a function of authors’ citing choices. We demonstrate that citation patterns can be largely accounted for by a memory process model in which small variations in the retrievability of scientific papers early on in their life cycle are magnified over time, resulting in the emergence of highly cited articles. This effect occurs even when there is no variation in the starting point citation probabilities owing to, say, quality or any other factor, and can easily produce so-called ‘super citations’ as a result of natural variation in the memorability of an article early in its life cycle. Our analysis casts doubt on the use of citation counts as a metric of research quality or impact and suggests that the number of citations garnered by papers and people may well be more reflective of the cognitive processes of the people doing the citing than of the author whose work is cited.

## 2. The exposure model of citation counts

Fundamentally, the exposure model treats citation behaviour as a memory-theoretic phenomenon. We assume that authors choose who to cite by retrieving candidate 'references’ from memory that are most relevant for the citing context. The citing context is assumed to act as a memory retrieval prompt for the author, bringing to mind the set of potential articles to reference in the text. In this sense, our assumptions about how and why particular papers are chosen by authors to cite are no different than any other act of memory retrieval, including, for example, choice among words when producing written documents. Thus, we make two core assumptions: (i) people choose what to cite based on what comes to mind in a particular context (i.e. context-dependent retrieval) and (ii) what comes to mind in a particular context is largely a function of factors that influence memory retrieval (e.g, encoding, frequency of exposure, recency of exposure, etc). Although the assumption that citation behaviour is a memory-theoretic phenomenon forms the basis of the model, it is not necessary to assume that all referencing decisions are made solely on the basis of retrieval of the reference per se, as we assume that the memory processes detailed herein broadly apply to how people generate search terms (from memory) when searching electronic databases and how they decide who to cite when evaluating the output of a search engine (i.e. by relying on familiarity or recognition of an author’s name). Nevertheless, we do assume that the majority of articles one decides to cite when writing an academic article are ones the author generates from memory or are chosen based on a recognition-based heuristic [31].^1^

The above two assumptions are motivated by a wealth of prior research in cognitive science, including work on the availability heuristic [32], the recognition heuristic [31], the validity effect, and the mere exposure effect [33]. The availability heuristic implies that the choices one makes (e.g. what to cite) in a particular context are largely driven by what is immediately retrievable from memory [32]. The mere exposure and validity effects reflect the fact that frequently or recently experienced items are preferred over, and viewed as more valid than, less-frequently or less-recently experienced items. Availability, the mere-exposure effect, the recognition heuristic, and the validity effect all derive from a common underlying memory mechanism accommodated by models of memory [34]. The common theme running through these phenomena is that retrievability functions as a cue for inferring other quantities [31]. For instance, prior exposure to causes of death in newsprint media or through one’s social circle predicts the perceived frequency of those causes [35,36]; perceived truthfulness of assertions increases as a function of repetition or recency [33]; and the perceived quality of products increases with brand familiarity [37]. Even the perceived quality of academic journals has been shown to correlate with the self-rated familiarity of journals [38]. As we show below, the two assumptions stated above are sufficient to account for the Matthew Effect in citation behaviour, in which articles that receive more citations are more likely to be cited again in the future [39], the finding that articles cited earlier in their life cycle are more likely to be cited more often [40] and the common belief that more highly cited articles are more important or of higher quality.

Before continuing, it is useful to point out that our proposed models are broadly consistent with a class of steady-state skew probability distribution functions [41–43]. This past work provides elegant mathematical frameworks for understanding the emergent properties of distributions that well describe citation frequencies. However, these frameworks do not provide a cognitive–theoretical account linking the distributions to plausible psychological mechanisms. Our approach is to model citation counts as reflecting properties of memory. Our conceptualization and theoretical framework derive from well-known memory mechanisms, reflecting the intuition that what authors decide to cite in many (if not most) contexts is, fundamentally, an act of memory retrieval. In this respect, our approach provides novel insights into the plausible behavioural and psychological processes that guide citation behaviour.

Broadly, we assume that the probability an article is cited is proportional to its exposure to the scientific community, where exposure is modelled as a function of how well an article has been encoded in memory. The quality of an article’s encoding can be assumed to be influenced by the number of times in the past the article has appeared in print, is cited in other articles, or is promoted in other ways (e.g. social media, blogs, presented at a conference, etc.). The model assumes that each article begins its publication life cycle with some units ( u ) of exposure owing to its publication. The probability that an article is selected for citation is simply the article’s accrued exposure units divided by the total number of exposure units across the set of hypothetical journal articles. Here, we implement the exposure model within the context of a simple model of memory. We illustrate that the natural stochastic properties of our model capture many of the properties observed in citation distributions, including the emergence of highly cited articles in the literature, without assuming that authors make citing decisions based on research quality or scientific merit.

We develop two versions of the exposure model: a stochastic-only version that merely assumes that the probability of citing an article is proportional to the number of prior exposures an article has received; and a memory-theoretic version that represents cited articles as vectors of features that are encoded in memory where forgetting can be modelled via a single parameter [44]. The stochastic-only model allows us to illustrate the properties of the model without imposing memory-theoretic assumptions. It makes minimal assumptions and has no free parameters. We assume that all articles in reference class ( R ) start on a level playing field ( u = 1), with an *a priori* equal probability of being cited ( p⁢(c⁢i⁢t⁢e⁢d)=u/∑u ). R defines the set of articles to be modelled. Articles in R will accrue a certain number of citations ( c ) within some predefined time frame, or epochs. Each epoch corresponds to a single opportunity for one article to be cited. Thus, the number of epochs is set to the total number of citations in R . Article citations are represented as frequency counts; thus, the stochastic model is essentially a frequency counter that tabulates how often each paper is cited over time. Articles selected for citation accrue additional exposure units randomly across epochs according to the iteratively updated distribution of citation probabilities. The value of u is incremented by 1 when an article is selected to be cited or receives other exposure during an epoch. Therefore, articles that receive more citations have a higher probability of being cited again, which in turn reduces the probability that previously uncited articles will receive future citations. Note that the model assumes that citations are a finite resource. This assumption follows from both practical constraints, i.e. there is no need to cite all possible articles to substantiate a claim, and from actual constraints many journals place on the number of allowable references in a paper. Thus, one consequence of the model is that the presence of highly cited articles within a particular distribution necessarily results in stagnation of citations for other articles.

To provide a concrete example of the model, imagine a set of 100 articles collectively cited 1000 times. Therefore, R=100 and the number of epochs is C=1000 . For each epoch of the simulation, one article from the field of 100 is selected at random to be cited. The probability of each article being cited in the first epoch is 0.01 ( 1/100 ). For the first epoch, the value of u for the cited article is increased by 1. The exposure probability of that article is then increased to 0.0198 (2/101), while the exposure probability for the non-cited articles all decrease to 0.0099 (1/101). During the second epoch of the life cycle, an article is selected randomly according to the updated probability distribution and the process is repeated. The entire process is repeated for c epochs to simulate the evolution of citation patterns across the life cycle of all 100 articles. Individuals interested in exploring the predictions of the model can access R code to run the model at https://osf.io/hmbup/ [45].

Note that the stochastic model has no free parameters and simulates the expected distribution of citations without the need for optimizing parameters to maximize fit. The assumption that all articles start with an equal value of u is obviously an oversimplification. It is easy to imagine how various factors could lead some articles or authors to naturally have greater exposure or receive greater attention than others. Nevertheless, we start with the assumption of a level playing field for the purposes of demonstrating the stochastic properties of the model.

It is also possible to implement the general processes detailed above within a more complex theory of memory. For this, we adopt an instance-based model of memory that represents experienced events as vectors of features [44]. The model starts with the assumption that each article in reference class R is represented by a single trace in memory. On epoch 1, a probe vector representing the citing context is created and matched against all traces in memory. This match yields an activation value for each trace, which is simply the cube of the similarity between the probe vector and trace. The normalized activations of all traces exceeding zero is then computed and a trace is sampled and retrieved probabilistically using Luce’s choice rule [46]. Because traces of references that have a greater similarity to the citing context are more highly active, they are more likely to be sampled and retrieved from memory and cited by an author. We assume that these new citations represent new exposure opportunities. Thus, for each newly cited article, a vector representing the newly cited article is added to memory and the process is repeated. Episodic memory therefore grows by one trace for each epoch of the model, with there being C+R total traces in memory at the completion of the simulation. The memory-theoretic version of the model is implemented in Python. A more detailed description of the model is included in appendix A. This supplemental material along with Python code for running the model is provided on [45]. Computer code used to distill the model output and produce the plots included in this article was written in R and is also provided at [45].

The memory-theoretic model has one parameter ( L ), which reflects the degree to which the traces in memory retain details of the original event. The L parameter can be thought of as capturing information loss that occurs either through lack of attention at encoding or via forgetting. Although encoding and forgetting are conceptually distinct, the model does not differentiate between these two types of information loss: both are operationalized as a reduction in features in the memory trace (see appendix A and supplemental code for details). Nevertheless, the model does allow us to explore the effect of information loss on citation patterns. The memory representations used in the memory-theoretic exposure model consist of vectors of features, which can be thought of as representing conceptual and contextual attributes of scientific articles. In principle, the similarity structure across populations of scientific articles can be modelled by representing articles that share conceptual and contextual attributes with vectors that share a high degree of feature overlap. To be sure, not only are the mechanisms involved with our memory-theoretic model consistent with theoretical models of decision making [31,47], it is also consistent with recent work showing biases in citation patterns arising from memory-related factors [48].

## 3. Demonstration simulations

To illustrate the properties of the memory-theoretic model, we conducted eight simulations varying the L parameter across four levels and similarity across four levels. The manipulation of L allows us to examine the impact of information loss on citation patterns. The manipulation of similarity allows us to examine whether citation patterns might be expected to differ between domains of research in which there is a high degree of conceptual overlap (e.g. domains in which the research community is narrowly focused) versus domains in which there is a lower degree of conceptual overlap (e.g. domains characterized by broad focus). It is well known that citation distributions show a strong positive skew, with very few highly cited articles and substantially more poorly cited articles. The question addressed by these simulations is whether the degree of skew, and hence the emergence of highly cited articles, might be affected by natural variability in information loss and/or domain similarity.

We chose a research ecology consisting of R = 739 articles that were collectively cited c = 57 310 times. This corresponds to the number of, and citations to, articles published in 1989 that were included in the database of articles (published in the psychological and brain sciences) previously scraped for statistical reporting errors [49,50]. We ran simulations to model information loss and similarity. To examine the effect of information loss, we varied L across four levels: L = 0.35, 0.55, 0.75 and 0.95, while similarity was held constant at S⁢i⁢m = 0.1. For reference, an encoding parameter of L = 0.35 corresponds to a case where 35% of the features corresponding to the original event are retained in the memory trace, whereas 65% are lost. A value of L = 0.95 corresponds to the case where 95% of an event's original features are preserved in memory and only 5% are lost. Note that the operation of L is stochastic, such that the actual degree of encoding for any individual trace will vary around its expected value. To model similarity, we constructed memory traces that share S⁢i⁢m percent of features, where S⁢i⁢m varied across four levels: S⁢i⁢m = 0.1, 0.25, 0.50 and 0.75. For these simulations, L was held constant at 0.50. Only a single simulation run was used for each value of L and S⁢i⁢m to more clearly illustrate the variation in predicted citation counts, as averaging across runs can mask this variability. Note that when S⁢i⁢m = 1.0, all traces are identical, when S⁢i⁢m = 0, they are all orthogonal. S⁢i⁢m values between 0 and 1 model gradations to which the traces in memory share overlapping features.

**Figure 1.**
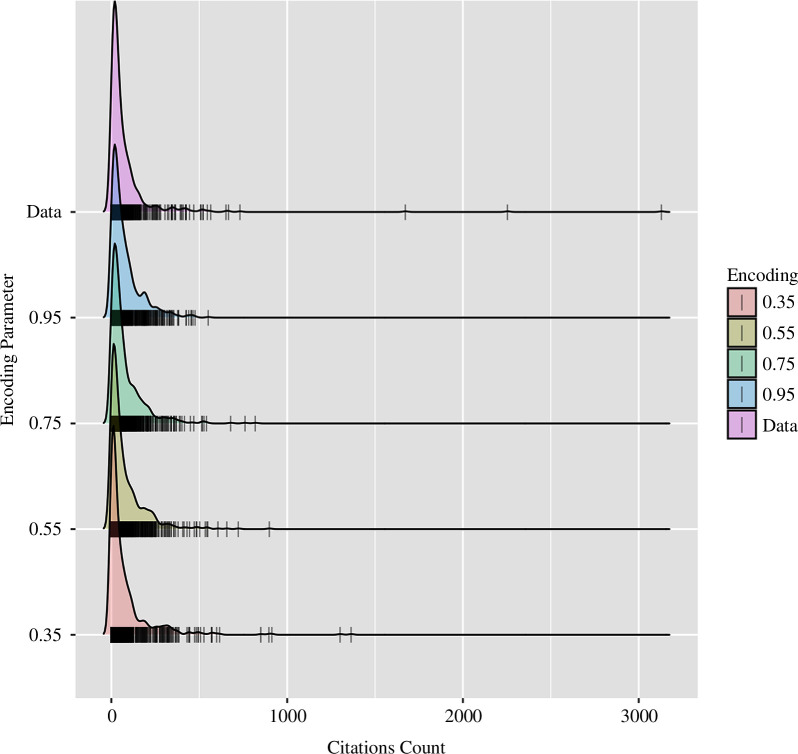
Density plots of the memory-theoretic model’s predicted distribution of citation counts as a function of the encoding parameter. The tick marks at the bottom correspond to individual articles.

**Figure 2.**
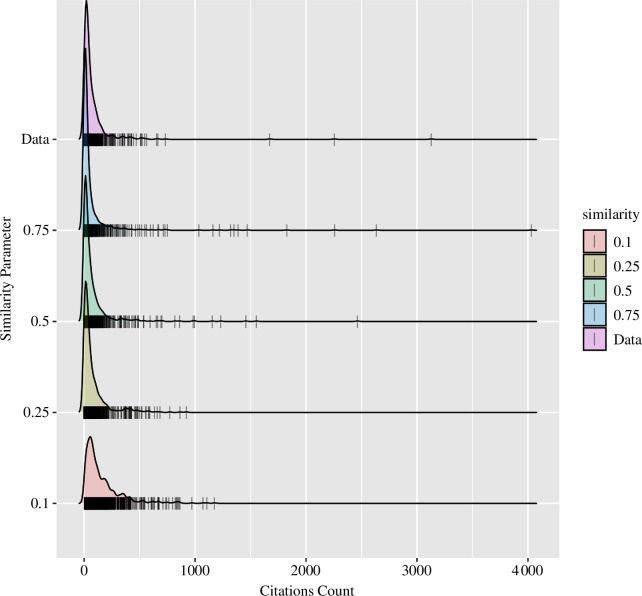
Density plots of the memory-theoretic model’s predicted distribution of citation counts as a function of the similarity of articles. The tick marks at the bottom correspond to individual articles.

Figures 1 and 2 provide the results of these demonstration simulations, along with the observed distribution of citation counts for the year 1989. A few observations are worth mentioning. First, decreased encoding increases the skew of the distribution. The model predicts that highly cited articles are more likely to emerge from an environment in which the expected information loss is greater. The effect manifests in the model based on natural variation in how well articles are encoded in memory. When most articles are poorly encoded, (low L ), the few articles that receive higher attention by random chance will have a greater retrieval advantage when compared to cases in which most articles are well represented in memory. For instance, when the expected value of encoding is 0.95, there is little room for articles to be encoded at a higher rate (only 5%). In contrast, when encoding is 0.35, there is a much greater opportunity for an article to be encoded at a much higher rate simply by chance. The disparity between the activations of the best and least-well encoded vectors should be greater when L is low.

The model also predicts increasing positive skew as a function of similarity: highly cited articles are more likely to emerge when the reference class consists of a set of highly similar articles. But, note also that high similarity also increases the number of *uncited* articles (see figure 2). These simulations suggest that research domains in which a lot of researchers are addressing the same (or highly similar) phenomena will produce highly skewed citation distributions with a small number of super citations or so-called ‘citation classics.’ Given the challenges with operationalizing similarity in real-world corpora that are tractable for our model, we have not examined these predictions in detail. In the simulations below, we hold similarity constant at a fairly low level ( S⁢i⁢m=0.1 ) and only manipulate L when fitting the memory-theoretic model.

## 4. Application of models to data

### 4.1. Data description

The goal of the modelling exercise was to evaluate the degree to which the model predicts the distribution of actual citations. The model does not enable us to predict the citation counts for individual articles, nor should it given that the underlying thesis is that citations derive from a stochastic memory-theoretic process. However, it does allow us to examine the degree to which observed distributions of citations follow the proposed exposure process. To this end, we are primarily concerned with the degree to which the characteristics of citation distributions are consistent with the exposure model.

The models were fit to R = 46 690 articles (cited C = 2 161 838 times) from 32 consecutive years (1985–2016) of articles included in the previously described database from the psychological and brain sciences [49,50]. The citation data for each of these articles was obtained using the scholar [51] package in R [52] and is available at [45], along with analyses for all 32 years of data. Citation data were gathered in January 2019 using the scholar API tool; citation counts for 3929 articles were checked manually by the first author to verify the accuracy of the automated tool. The correlation between the automated and hand-checked values was almost perfect (*r* = 0.99), and any deviations were due to cases in which papers accrued additional citations between the time that the automated values were obtained and the counts were checked manually.

We ran the stochastic version of the model for 20 repetitions to simulate the distribution of citations for each year. We applied the memory-theoretic version of the model to each year, wherein we: (i) varied the model’s encoding parameter, L , and conducted 10 repetitions at each value of L , and (ii) manipulated L across a minimum of four levels in increments of 0.1 to find an approximate best fit. A total of 144 simulations were run across the 32 years of data, with 10 repetitions of each simulation. Note that the models produce highly consistent predictions across simulation runs, with pairwise correlations between runs ranging from 0.97 to 0.99 for a given value of L . This is largely due to the fact that the citation probabilities converge on a steady state. Thus, there is little to gain by running additional simulations. The memory-theoretic version of the model is highly computationally intensive, as its memory computations are repeated for each epoch, with one new trace added to memory for each epoch. For our application of the model, the median number of epochs for a single simulation run was 66 769. Thus, a single simulation run entails a median of 66 769 iterations of the model, each of which was repeated 10 times for each value of L. For all simulations, the value of S⁢i⁢m was held constant at 0.1. We did not attempt to fit distributions by varying S⁢i⁢m .

We use two indices to assess model fit (i.e. to characterize the overlap between the predicted and actual citation distributions). One index was the A statistic, which is a non-parametric probabilistic measure of effect size. The value of A reflects the probability that a randomly drawn value from the observed distribution is greater than a randomly drawn value from the predicted distribution. A is a non-parametric estimator of the Common Language effect size and has an expected value of 0.50 under the null hypothesis that two distributions are identical. The A statistic is closely related to both the Mann–Whitney U and Kendall’s tau, with the U statistic providing a valid test of the null hypothesis. The second index was a measure of the degree to which the probability density functions (PDFs) between two distributions overlap [53]. This is referred to as the overlapping index. We use the gemmR package [54] to compute A and the overlapping package [55] in R to compute the similarity between two PDFs. Note that both the expected overlap between any two PDFs generated from the same population and the expected theoretical upper bound for overlap between two empirical PDFs with the same population parameters is sample size dependent. This upper bound can be estimated by simulating two distributions with sample size N drawn from the same population and computing the overlap between the two densities. The upper bound was estimated from all pairwise runs of the simulation. There were 20 * (20 − 1)/2 = 190 pairwise comparisons for each simulated year for the stochastic model. There were 10 * (10 − 1)/2 = 45 pairwise comparisons for each simulated year for the memory model. The average overlap for the stochastic model distributions ranged from 95.53% to 97.78%. The average upper bound for the memory model ranged from 94.95% to 97.83%. Thus, we provide both the raw and the adjusted value of overlap corrected for chance. The adjusted overlap is computed by dividing the observed overlap by the mean simulated upper bound for each sample size.

### 4.2. Simulation results

We fit the simulation data separately to each of the 32 years of citation data. To illustrate the fit of the model, we provide the average fit across all 32 years and highlight three specific years that represent the full range of fit—from the year in which the models fit the worst to the year in which the models fit the best. These three example years were chosen based on the fit of the stochastic model.

**Figure 3.**
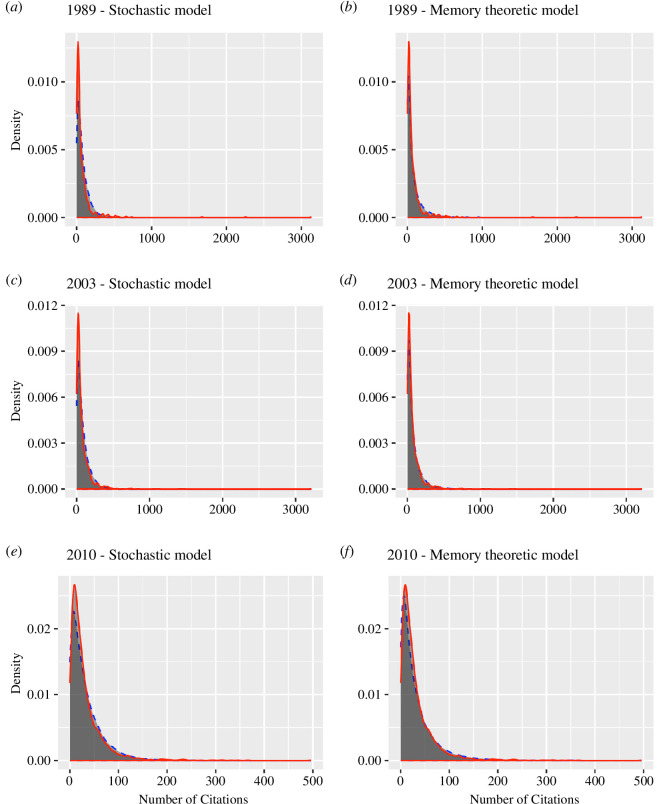
(*a*–*f*) Density plots of actual citations (red) and model predictions (blue) for 3 years of publications. Model predictions are based on the median value of each quantile across 100 simulations for each year.

Panels *a–c* of figure 3 plot the actual and predicted distributions of citations using the stochastic model. The 3 years that are plotted, 1989, 2003 and 2010, represent the years for which the stochastic model had the *poorest* fit (1989), *median* fit (2003) and the second *best* fit (2010) based on the overlapping index. While the year 2016 had a slightly better fit than the year 2010, we chose to plot the data for 2010 instead of 2016 because 2016 included a much smaller number of both articles and citations than the several years prior. Data for 2016 were included in the calculation of the average across all 32 years. The average of 2016 fits, as well as all other years, are available on OSF. Table 1 provides the fit statistics for these 3 years as well as the average fit across all 32 years. The average adjusted overlap for the stochastic model was 91.1%. The mean value of A was 0.546. Both these metrics indicate that a large proportion of the variability in true citation counts is consistent with a simple stochastic process without assuming any specific psychological mechanisms. However, the model’s fit is far from perfect. A version of this model has been used to describe word frequency distributions in written text [43]. As much as word usage involves retrieval, it is plausible to assume that the data-generating mechanism that produces word-frequency distributions is similar to the data generation underlying citation distributions.

**Table 1.** Average fit statistics for three representative years of article citations and the mean of all 32 years of citations. The years were selected to represent a year with poor fit (1989), a year with median fit (2003) and a year with one of the top fits (2010), based on the stochastic model and using the *A* statistic as the index of fit. R = number of articles; C = number of citations; A = non-parametric measure of effect size; O⁢V = uncorrected overlap of real and simulated citation distribution; $O\,V_{a\,d\,j}$ = overlap of real and simulated citations corrected for chance.

year	R	C	A	OV	OV_adj
stochastic model					
1989	739	57 310	0.578	82.43	85.71
2003	1131	92 948	0.547	87.67	90.68
2010	2128	67 212	0.509	92.57	95.03
mean 32 years	1459	67 557	0.546	88.02	91.11

memory-theoretic model					
1989 (*L* = 0.55)	739	57 310	0.504	91.38	96.08
2003 (*L* = 0.75)	1131	92 948	0.504	93.49	97.65
2010 (*L* = 1.0)	2128	67 212	0.523	93.47	96.02
mean 32 years	1459	67 557	0.510	92.47	90.54
minimum 32 years	694	39 182	0.502	90.26	93.45
maximum 32 years	3874	1 07 733	0.537	94.81	98.77

Panels *d*, *e* and *f* of figure 3 plot the actual and predicted citation distributions based on our memory-theoretic account for the three selected citation distributions used to demonstrate the stochastic model. The bottom half of table 1 provides the summary fit statistics for these distributions along with the mean, minimum and maximum values across all 32 years. Overall, the memory-theoretic account provides a better fit to the data than the stochastic model, as illustrated by the average values for A and the overlapping indices. Recall that the A statistic is an index of the degree to which two distributions differ, analogous to Cohen’s d but expressed as a probability. Under the point-null hypothesis of no difference, A = 0.50. By comparison, Cohen’s d = 0.10 is roughly equivalent to A = 0.528 [56]. The mean value of A across the 32 years was 0.510, indicating that the simulated and actual citation distributions are nearly identical. Indeed, a comparison of the observed distribution with the memory-theoretic predicted distribution showed strong support for the hypothesis of no difference for most years (median $B\,F_{01}=14.9$ in favour of the null). For this analysis, we used a non-parametric Bayes factor based on the Mann–Whitney U statistic [57]. In only 3 of the 32 cases was there evidence for the null inconclusive ( $BF_{01}<3$ ), and in only one case (2010, $B\,F_{01}=0.66$ ) was the $B\,F_{01}$ less than 1.0.

Although the model provides an excellent fit to the data, two aspects of the distributions are not well accounted for: the model underpredicts a small number of papers with extreme citation counts and overpredicts the number of papers that will be uncited. Although it is possible for the memory-theoretic model to better account for highly cited articles in the extreme tail of the distribution by decreasing the encoding parameter or varying the similarity of the vectors, doing so further increases the predicted proportion of uncited papers.

**Figure 4.**
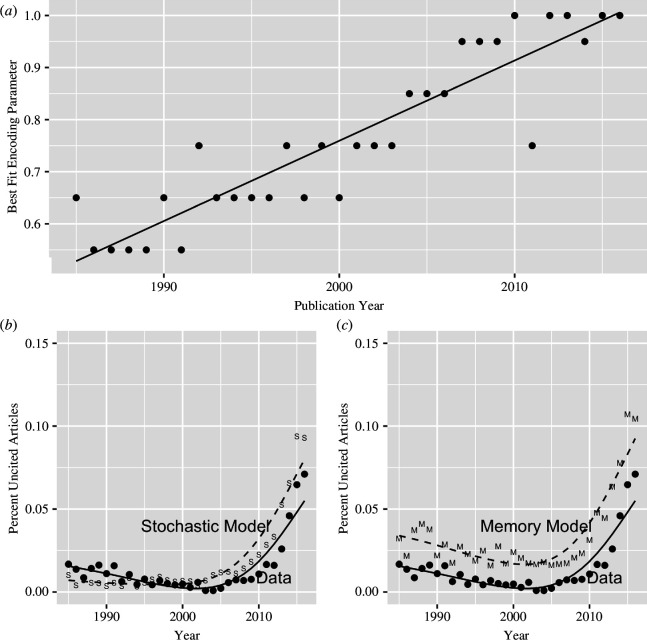
Plots of best fitting encoding parameter as a function of publication year (*a*) and predicted number of papers with zero citations for both the stochastic model (*b*) and the memory-theoretic model (*c*).

As shown in panel *a* of figure 4, the value of L for the best-fit models generally increased across years, with the higher values of L needed for modelling citations to articles published more recently, and lower values of L for articles published in the more distant past. This pattern is consistent with the memory-theoretic interpretation of citation behaviour, as we assume papers published further in the past are less well-represented in memory due to information loss.

Panels *b* and *c* in figure 4 provide a more detailed examination of the model’s predictions in regard to uncited articles. Panel *a* plots the best-fitting value of the encoding parameter for each publication year. The positive slope of the best-fitting line can be interpreted as reflecting greater forgetting over time. For instance, L is lower for articles published in the 1980s compared to the 2000s, capturing greater information loss of publications further in the past. Panels *b* and *c* plot the actual and predicted proportion of uncited articles for both the stochastic model (panel *b*) and the memory-theoretical model (panel *c*). Overall, both models appear to overpredict the proportion of uncited articles. Although the stochastic model better accounts for the proportion of uncited articles in the 1980s and 1990s, it fails to accurately capture the non-monotonic function observed in the data.

**Figure 5.**
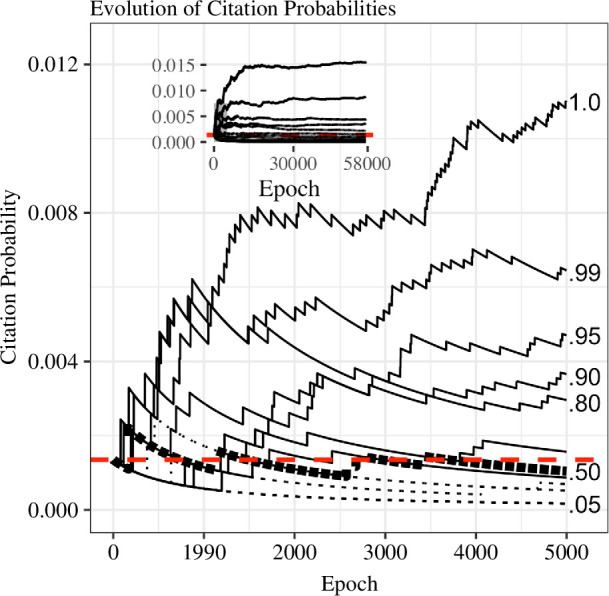
Plot of the predicted evolution of citation probabilities over time for the memory-theoretic model for year 1989. Main panel shows first 5000 epochs. The inset shows evolution over all epochs. Each line represents a different quantile (1.0, 0.99, 0.95, 0.90, 0.80, 0.70, 0.60, 0.50, 0.40, 0.30, 0.20, 0.10, 0.05, 0.01 and 0.00) based on the final probabilities. The thick back-dashed line corresponds to the 50th percentile of the distribution. Quantiles ending above 0.5 are represented by solid lines, whereas those ending below 0.5 are dotted. The red-dashed line corresponds to the starting point probabilities.

An interesting question concerns why some articles accrue more citations than others. Prior work shows that articles cited *earlier* in their life cycle tend to be cited more often *across* their life cycle compared to articles that receive their first citation later [40]. This general trend is naturally captured by the exposure model, as shown in figure 5, which shows the evolution of citation probabilities for 11 simulated articles drawn from the simulation of citation distribution for the year 1989. The numbers to the right correspond to quantiles of the final predicted citation count. For instance, the article with the highest final citation count is given by the uppermost line (1.0), and the article corresponding to the 99th percentile in terms of final citation counts is given by the next highest line. The inset of this plot shows the evolution of citation probabilities predicted by the memory-theoretic model across the entire lifespan; the main figure shows the citation probabilities for the first 5000 Epochs of the model.

A few patterns can be discerned from these plots. Consistent with past findings, the most highly cited articles are those that are cited early in their life cycles [40]. This pattern is important for at least three reasons: first, it indicates that highly cited articles are guaranteed to emerge naturally from a stochastic system, even without assuming that highly cited articles are any more meritorious than less-widely cited articles. Second, the simulation suggests that interpretations of early citation count as an indicator of long-term citation ‘impact’ is a self-fulfilling prophesy. As the model demonstrates, papers that are cited early will naturally accrue more citations simply owing to the stochastic nature of the model and the increased exposure that they enjoy. Third, to a large degree, what determines whether an article becomes highly cited can be plausibly accounted for by random variability.

A second pattern that can be discerned is that although early citations do not guarantee that an article will become highly cited, the model anticipates that the lack of early citations can make it very difficult for meritorious ideas to emerge later. Put another way, with each passing epoch in which an article goes uncited, the odds that it will ever be cited decrease. Although the model anticipates only a small percentage of papers will go uncited, it clearly shows that these papers become increasingly less likely to be cited with time. The implication of this effect is that some articles, and by extension the scientific knowledge contained within those articles, are stymied by a small number of highly cited articles.

### 4.3. Super citations

The simulations presented in the left column of figure 3 and the top half of table 1 assume that all articles start with an equal probability of being cited. As discussed above, however, there is plenty of evidence to suggest that the playing field is uneven and that some articles (or researchers) enjoy an exposure advantage that heightens the amount of attention their work receives. This advantage may be due to the topic (hot topics are cited more frequently because there are more people working on the problem), the size of one’s academic or social network, the use of social media to promote one’s work (preprint servers, tweeting, etc) or merely the readership of the journal. While it is also entirely possible that buried among the factors affecting exposure are attributes related to scientific merit, it is important to note that: (i) scientific merit is neither necessary nor sufficient to ensure exposure and (ii) there is little evidence to support the assertion that citations are related to merit or quality [21,30]. This general pattern in which the occurrence of highly cited articles simultaneously increases the number of poorly cited articles defines the Matthew Effect [39] and has been observed in analyses of citation patterns for National Academies members [58].

The above results indicate that small changes in the starting point exposure probabilities are sufficient to create highly cited articles. At the same time, the simulations illustrate that articles that benefit from increased attention early on detrimentally impact citations to other papers. Thus, not only do the rich get richer, but the poor get poorer, as was originally observed by Merton [39]. Our memory-theoretic model counterintuitively predicts that the Matthew Effect is exaggerated under conditions in which there is a relatively high degree of information loss (i.e. poor encoding, as modelled by lower values of L ). Although it is possible that at least some of these super citations do indeed reflect transformative or paradigm-shifting contributions, the direction of causality has not been established. For instance, do articles become transformative because they are cited and propagated in the literature due to the Matthew Effect [39], or do they become highly cited because of their ideas? Because articles that are uncited are by definition less likely to have been read by other scientists, it is impossible to assess whether citation counts themselves are diagnostic of the merit of the ideas contained within articles.

## 5. Discussion

The primary finding derived from our simulations is that highly cited articles can arise from a noisy memory retrieval system characterized by information loss. Indeed, the more noise there is in the memory system, the more likely it is that highly cited articles will emerge. The second finding is that a relatively small amount of variation in exposure of an article early on in its life cycle can create a relatively large variation in future citation counts. Our findings demonstrate that random variation in citation patterns early on has an out-sized effect on future citation counts by altering the exposure probabilities—a finding that is consistent with past research on citation patterns [40] and easily explained as a function of memory theory. Moreover, the simulations offer an alternative account for citation patterns that challenge traditional orthodoxy that citations reflect the underlying scientific merit of the research. Specifically, our simulations illustrate that the pattern of citation distributions can be largely accounted for by recasting authors’ decisions about what to cite as an emergent property of memory retrieval. This account of citation behaviour suggests that the critical variables underlying whether a paper is cited frequently or less frequently are largely determined by variables that affect its memorability. We suggest that many of the variables previously identified as related to citation patterns (e.g. length of title, familiarity of the authors, ‘surprisingness’ of findings, use of social media to promote ones work) are in fact variables that affect memory retrieval. Not only does this explanation make intuitive sense, but it is also consistent with a variety of findings within psychological science, such as the mere exposure and validity effects, as well as recent work showing that citation counts are sensitive to memory-related factors [48].

The idea that citation patterns follow a stochastic process is consistent with prior work [58]. Citation patterns and the apparent positive relation between the number of publications one has and the occurrence of highly cited articles [58] are also consistent with the exposure model. Mechanistically, our model assumes that this stochastic process is rooted in memory processes, such that the more available a reference is in memory, the more likely it is to be retrieved and cited when cued by the citing context.

The exposure model clearly challenges the common belief that citation counts reflect the underlying scientific merit of the cited work. Unfortunately, the emerging literature examining the relationship between scientific quality and citation counts has largely failed to find consistent relationships between measures of research quality and citation counts [11,21,22,24,25]. In their review of the literature on this issue, Aksnes *et al.* [30] concluded that ‘citation indicators seem of little help in the evaluation of the solidity/plausibility, originality, and societal value of research.’ (p. 12) At the same time, there is evidence that citation counts are related to a variety of non-quality-related factors, including some that directly reflect the exposure of an article to the academic community.

Note that the choice of what is retrieved is based on the similarity between the citing context and the traces in memory. Thus, the model will be biased (in a good way) to select references to cite that have a strong match (high similarity) to the citing context. One implication of this feature is that articles written on so-called ‘hot topics’ will necessarily have a heightened probability of being cited relative to articles on less-popular topics. This is a natural by-product of using the citing context as the probe: If a particular context (e.g. on a popular topic) occurs more frequently, traces of articles that match that context are more likely to be retrieved from memory.

### 5.1. Implications

An important question raised by our model concerns the factors that might affect the exposure probabilities of one’s work. Although there are a few seemingly obvious candidate hypotheses, we suspect that what drives exposure probabilities is complex and multiply determined. Obvious sources of exposure include the size of a journal’s readership, social media, press releases, news media reports, open-access and use of preprint servers. However, other factors likely enhance the visibility and exposure of a paper, including name recognition [3], and pressure to cite colleagues, competitors, mentors or others within one’s social or research network.

While the results provide a recipe book for increasing one’s citation counts, they should also stimulate concerns about factors that might limit the exposure of one’s research to the scientific community. One such concern is parenting. Women are far more likely to be the primary caregiver for young children and as a result forego conference attendance during critical stages in their careers [59]. Because conferences provide an important opportunity for researchers to promote their latest research findings to the research community, missing these opportunities can negatively impact the visibility of these scientists [5,60]. The same could be said for scientists who forego conference attendance in order to reduce their carbon footprint. The exposure model anticipates that these early missed opportunities can be magnified over the course of one’s career and affect citation rates. It’s also useful to point out that the model suggests that whether or not a particular paper is cited is not just a function of its own exposure, but also a function of the exposure of other papers. That is, the probability that a paper is cited will decrease over time as a function of the increased exposure of other papers. In other words, in the marketplace of scientific ideas, what may matter most is how much effort is put into promoting one’s work, not the merit of the idea being promoted. In regard to promotion and tenure policies or practices that weigh citation counts heavily, this is clearly problematic.

Our simulations assume that articles and researchers all start on a level playing field. But this is not the case in real life. It is easy to imagine variables that advantage some and disadvantage others: do researchers from more prestigious institutions receive greater exposure to their work, or does it receive more attention, simply owing to their affiliations? Does co-authoring with an already well-known researcher increase the attention the article receives? Might subtle racial, gender or other biases alter the exposure that authors receive in the media and affect citation patterns [61,62]? Might the cultural climate of a scientific discipline in terms of gender, racial, or even political inclusivity affect researchers’ willingness to attend conferences to present their work? Not only is it easy to imagine cases of each of these scenarios, but also evidence suggests that such effects are plausible. Decades of work on the mere exposure effect shows that people have a preference for familiarity and even believe more strongly in claims deemed more familiar [33]. Work on racial prejudice has documented evidence of racial preferences in citation patterns [61,62]. The immediate effects of any one factor may not be particularly large, but the effect does not need to be large to have a major impact across the life cycle, leading to large systematic biases [18–20]. Small changes in starting values at the outset of one’s career or in the initial phases post-publication are easily magnified over time. There is clearly variation to be explained in citation counts, and aspects of the quality of the research may well be one small source of variation. However, what is clear from the simulations is that one need not assume that citation counts are based on quality to observe large disparities in citation counts.

Our simulations have a clear connection to prior work on the effect of perverse incentives on research quality. Recent work has illustrated that incentives that focus on maximizing publication quantity can result in the selection of poor scientific methods [63]. This selection process can happen in two ways. First, increasing the number of research products necessarily comes at a cost [64]. As shown by Smaldino and McElreath [63], those costs can manifest in poor research methods and ultimately lead to the selection (hiring, tenure and promotion) of researchers who use substandard research practices. Second, as the number of publications one produces increases, the chances that one of those articles becomes highly cited also increases—merely as a function of chance. This later prediction is a natural consequence of the exposure process described above and is consistent with previous findings [58]. Thus, not only will researchers who publish more look more productive (in terms of quantity), but they will also benefit from greater citation counts and a higher chance of at least one paper becoming a super citation. By this account, one’s citation ‘impact’ has more to do with chance, than it does with the merits of one’s work.

One potential criticism of our work is that perhaps the articles that are cited early on in the life cycle are chosen for their overall quality. If this were indeed the case, then one would expect that an article’s future citation counts would correlate with objective measures of quality. Unfortunately, there is little to no convincing evidence that this is the case: the evidence linking citations to objective measures of quality, and even subjective measures of quality, is weak at best [21]. However, as discussed above, there *is* evidence that citation counts relate to non-quality factors, many of which affect an article’s ‘visibility’. The prima facie argument that people cite articles based on quality does not have much support in the literature [26].

Our model offers a plausible competing account of citation patterns rooted in memory theory. Of course, even within this mechanistic account, one can still postulate that how well an article is represented in memory is due to some aspect of quality. Although we cannot fully reject this possibility, it is clear to us that research quality is neither a necessary nor sufficient factor in determining whether an author will retrieve a particular citation in a given context. One interpretation of our model’s predicted citation distributions is that they represent the expected distribution of citations under the null hypothesis, where the null here is defined as a probabilistic data generating mechanism. Regardless of whether one maintains the belief that citations reflect quality or impact, the fact that the true distribution of citations is indistinguishable from a probabilistic process should give us pause and lead us to question the discriminate validity of citation counts. The standard in classical statistics is to infer the presence of an effect by comparing the observed data to a properly formulated null hypothesis. That same standard should apply to the use of citation counts to measure scientific merit, impact or research quality.

Rather than measuring quality, a natural response to our conclusions could be to assert that citations reflect influence or ’impact.’ The problem with this response is that it creates a potential tautology in which the very thing we are trying to measure is the definition of the measured variable. Citations cannot be used both to predict impact (as the predictor variable) and as our measure of impact (as the dependent measure). Even if one dismisses the tautology, redefining citations as a measure of impact does not actually solve the central problem. Whether one believes citations reflect quality, ‘impact’ or something else, their use as a selection variable is not justified if it is indistinguishable from a random process (as our model results imply) or largely determined by a host of non-merit-based variables (as implied by the extant literature). Social influence, as is produced by sharing work on social media, preprint servers, publishing with established researchers, and promoting work in other ways should not play a role in evaluating science. Put more bluntly, we suspect that most people would object to the use of any variable to select who to hire or promote that is either random or driven by extraneous factors.

### 5.2. A path forward

If citation counts and impact factors are to be abandoned, how might one evaluate the quality and impact of scholarly work? A number of proposals have been put forth that we believe provide a viable set of responsible indicators for assessing scientists (RAIS’s [17]). Others have summarized a proposal that involves serious evaluation of a small set of a researcher’s best research products [17,65] and others suggest the inclusion of criteria that capture one’s use of research practices that promote reproducibility (making research tools, analysis code and data publicly available); Fraley and Vazire [66] proposed the N-pact factor—a metric for assessing journal quality based on statistical power or sample size. In addition to these, and in domains where this is feasible, we suggest conducting reproducibility audits of scientists’ research as a way of verifying and scoring one’s use of open science practices, while asking for authors to more fully annotate their CVs to document efforts undertaken for each article that support reproducibility [16]. In contrast to research metrics that emphasize quantity (quantity of publications, quantity of citations, high impact factors), which can promote the use of poor scientific methods [63,64], the alternative metrics proposed above are designed to incentivize what the scientific community values: quality science based on sound methodology whose conclusions can be trusted. More than that, because science is a public good often funded by taxpayer dollars, we should strive to maximize the benefit of our science to the public. Doing so requires sharing our research in the broadest way possible to both facilitate future science and make the research products available to the general public.

In sum, it is time to stop pretending that citation counts are a reasonable measure of the quality of a scientific paper or researcher. Although one cannot reject quality as one of the possible variables that affect the exposure probabilities of a paper, there is sufficient evidence that citation counts are multiply determined by a host of factors unrelated to scientific quality. These factors are likely to drown out any signal due to quality. Indeed, the fact that highly cited articles can emerge from processes early on in the life cycle of an article should give us pause when considering these numbers for tenure and promotion decisions. In as much as citations reflect an exposure-like process, their use for evaluating scientists favours *exclusivity* over *inclusivity* by disadvantaging those who have historically lacked a voice in science.

## Data Availability

Data are archival data curated from distributions of citation counts of 46 690. These data are publicly available. The curated data as well as all code and simulation models are available at https://osf.io/hmbup/ [66].

## Footnotes

^1^
Electronic searches can amplify previously highly cited articles even more because search databases may sort potential references by citation counts.

## Appendix A.

To further test our memory-theoretic approach to studying citation counts, we built a memory model to formally inculcate citation behaviour in the context of memory processes. This model was an adaptation of the MINERVA 2 model of cued recall from long-term memory [44]. Specifically, citation behaviour is characterized as a form of cued recall, where the writing context serves as a cue used to probe memory. Articles were recalled with respect to a similarity-graded activation mechanism. Note that this approach makes the same assumptions as the exposure model: (a) citations are selected as a consequence of what comes to mind in a particular context and (b) what comes to mind in a particular context is a by-product of memory processes.

### A.1. Model

The model represented articles as episodes in memory, each abstracted in the form of one feature vector where the value of a unit in that vector was randomly drawn from whole numbers between -1 and +1 . The imperfect nature of learning was simulated by degrading the representation of each event vector in memory. This was achieved by reverting the value of each unit in the vector to 0 with a probability of 1-L . Thus, the presence of 0 in an event vector can be interpreted either as the absence of a feature or, after the implementation of the encoding parameter L , as a failure to encode a feature to its representation in memory. Traces, therefore, consist of a vector of −1’s, +1’s, and 0’s, with the proportion of 0’s increasing as L decreases. As an example, a single trace is represented as [−1,0,+1,0,−1,−1,+1,0,−0,−1]. The vector length is of a fixed length.

Context probes, like traces in memory, were randomly generated vectors of equal length to the event vectors, where each unit was drawn from whole numbers between -1 and +1 . The activation of each event in memory depended on its similarity to the context probe. Similarity was computed with equation (A 1), where similarity of the trace ( $S_{i}$ ) is the mean product of each feature in the probe vector ( $P_{j}$ ) and the corresponding, non-zero feature in the i th memory trace ( $T_{i\,j}$ ).

(A 1)
$$S_{i}=\,\sum_{i=1}^{n}P_{j}\,T_{i\,j}\,n$$

(A 2)
$$A_{i}=S_{i}^{2}$$

As in MINERVA 2, the signal-to-noise ratio was boosted by raising similarity to the third power, ensuring that memory traces that most resemble the probe stand out against those that do not equation (A 2). Traces with negative activation were then set to 0 prior to computing the intensity ( I ) of memory equation (A 3), which accounts for the total amount of activation across all traces in memory ( M ) in response to the context probe.

(A 3)
$$I=\sum_{i=1}^{M}A_{i}$$

(A 4)
$$P\,\left(T_{i}\right)=\frac{A_{i}}{I}$$

Articles were selected for citation with respect to their activation in memory. This was formalized by implementing Luce ′ s choice axiom [46], such that the probability of the i th trace being selected ( P⁢(T⁢i) ) was equal to the ratio of its activation and intensity equation (A 4). Once selected for citation, an additional trace was added to memory for the selected article. The original event vector was encoded to that trace as was done at the start of the simulation, where each unit in the vector could be replaced with a 0 with a probability of 1-L . This process continued until a previously determined number of citation events occurred. The frequency with which an article was cited in a simulation was determined by the number of times it had been encoded in memory.
